# Head and Neck Plasmacytoma With Diffuse Amyloid Deposition: A Case Report

**DOI:** 10.1155/crid/5576158

**Published:** 2025-11-04

**Authors:** Amirah Alnour, Mohammad-Aasem Abbas, Ameen Rahmoun, Zein Ibrahimbasha, Lana Sayal, Marco Isaac, Khaldoun Darwich, Zaven Karabet, Anas Abdo

**Affiliations:** ^1^Oral and Maxillofacial Pathology Department, Damascus University, Damascus, Syria; ^2^Oral and Maxillofacial Surgery Department, Damascus University, Damascus, Syria; ^3^Anatomic Pathology Department, Damascus University, Damascus, Syria; ^4^Oral Medicine Department, Al-Wataniya Private University, Hama, Syria; ^5^Oral and Maxillofacial Radiology Department, Delta University, Dakahlia, Egypt; ^6^Aqaba Medical Sciences University, Aqaba, Jordan; ^7^Restorative Dentistry Department, Damascus University, Damascus, Syria

**Keywords:** amyloidosis, mandible, myeloma, plasmacytoma

## Abstract

A variety of carcinogenic agents and the innovation of diagnostic methods have a huge impact on the biological profile of numerous tumors over time which reflect in their diagnosis and behavior. The ability to predict the tumors' behavior, mainly the malignant ones, and develop personalized therapy could change the lifestyle of the patients. The unpredictable histopathologic profile of plasmacytoma, for example, and its linked clinical behavior suggest that the fluctuations are linked to its behavior and prognosis as well. We introduce in this case a unique type of plasmacytoma with valuable clinical data and follow-up that may support literature with conspicuous documented cases of this malignant tumor. Indeed, this entity has obvious invasive and destructive features and yet a good prognosis. A good understanding of this malignancy will extremely enhance the long-term healing of the patients. This paper presents a destructive plasmacytoma with peculiar histopathologic features and yet has a good prognosis during our follow-up. We observed a multinodular mass in the right mandible extending above the sternocleidomastoid muscle in a 58-year-old female patient who was referred to the oral and maxillofacial department in the faculty. After half-mandible excision and right neck resection, the lesion was diagnosed as a plasmacytoma with amyloid deposition, and the patient was referred to an oncologist. And the patient is still under follow-up without any recurrence. Despite the low risk of extramedullary plasmacytoma progressing into multiple myeloma, the clinical management is still a challenge because of the potential for recurrence. Understanding the behavior of this tumor and its management strategies is crucial to obtain the best prognosis for the patients.

## 1. Introduction

Epidemiology: Plasmacytoma is a rare entity. It is a malignant tumor consisting of atypical plasma cells that can arise either in bone or in soft tissue. It is divided into solitary plasmacytoma of bone (SPB) and extramedullary plasmacytoma (EMP). SPB presents as a discrete mass associated with pain and bone destruction; 50% of solitary plasmacytoma develop multiple myeloma (MM). On the other hand, EMP is a solitary tumor composed of abnormal plasma cells that present in the soft tissue; 15% of extramedullary patients develop MM [1, 2].

Clinical relevance: Plasmacytoma usually presents with idiopathic anemia, deep bone pain, increased monoclonal proteins in urine and serum, and hypercalcemia. SPB presents with pain. Occasionally, it occurs in the skull as exophytic mass and associated with headaches, diplopia, and dizziness. It is not uncommon to see pathological fractures because of SPB infiltration and the damage of soft tissue.

Eighty to ninety percent of EMP occur in the head and neck region and aerodigestive tract, mainly in the oral cavity and sinonasal tract. They may present with headache, nasal obstruction and discharge, dysphagia, sore throat, epistaxis, and diplopia. It is uncommon to involve larynx and gastrointestinal tract [3].

Diagnostic challenges: The plasmacytoma has three infiltration cells: nodular, interstitial, and diffuse. In the nodular pattern, the plasma cells distribute between the hematopoietic cells as clusters of cells. While in the interstitial pattern, the plasma cells invade small spaces in between hematopoietic cells. This makes the diagnosis by traditional stains a real challenge, and the use of immunohistochemical stain is essential to reach a final diagnosis [3].

The cancerous plasma cells themselves have four shapes: the mature, immature, pleomorphic, and plasmablastic types. Generally, plasma cells appear as round cells with eccentric nuclei. The Hoffa clear zone next to the nucleus represents a prominent Golgi apparatus, which is involved in immunoglobulin production. Occasionally, immature plasma cells with nuclear pleomorphism and binucleated/trinucleated cells can be seen [3–5].

Immunohistochemical stains for this tumor include diffuse positivity for CD138, which highlights the plasma cells. While plasma cells express positive stain for CD138 antigen, a slight decrease in CD38 is accompanied. Further amelioration of plasma cells can be detected using CD45 and CD19 antigens, especially for abnormal plasma cells. These diverse expressions of varied surface antigens aid in orienting the identification of the normal plasma cells from the abnormal ones during different stages of the myeloma. Additionally, positivity of a single chain immunoglobulin in IHCs confirmd the diagnosis [6].

Amyloid deposition–associated cases: One of the intriguing things in plasmacytoma is the deposition of amyloid in various tissues and organs. This deposition occurs particularly in bone myeloma, while large amyloid deposition may occur in connective tissue adjacent to the bone lesions [7, 8]. Amyloid is an abnormal extracellular protein that can be seen in various tissues in accordance with amyloidosis or associated with specific diseases such as myeloma. Under a microscope, it appears as amorphous acidophilic material in traditional stains (hematoxylin and eosin). One of the most common dyes that is used in amyloid detection is Congo red. Congo red–stained amyloid is seen by fluorescent microscope (FM), in which the amyloid will appear apple-green birefringence by polarized light [9, 10].

Treatment: Usually in solitary plasmacytoma, the patients undergo a complete or partial surgical removal, which remains the first choice in management. Because of the relatively high recurrence rate, radiotherapy is recommended after the surgery. The consensus on the optimal dose of treatment reaches 40–50 Gy over 1 month. This dose is divided into daily exposures of nearly 1.8–2.0 Gy. The involved tissues are measured radiographically before to assess 2 mm of normal, intact tissue, while the efficacy of adjuvant chemotherapy is still under debate [11].

## 2. Case Presentation

**Figure 1:**
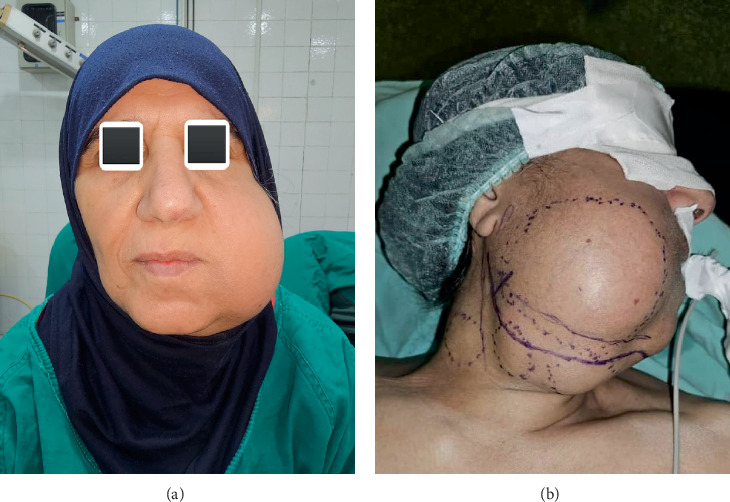
(a) A 58-year-old female patient complaining of right mandibular swelling that extends toward the carotid triangle. (b) Preparation for the surgical treatment.

A 58-year-old Syrian woman was referred to the oral and maxillofacial department in the dental college of Damascus University complaining of an old mass in the mandible that had increased in size gradually during the last 5 years and extended to the neck (Figure 1). The main complaint was difficulty opening the mouth with limited pain and discomfort. Extraoral oral examination revealed deformity in the face resulting from tumor mass that expanding the cortical bone of the right maxilla. There was a mass in the mandible and another one extended along the sternocleidomastoid muscle. Incisional biopsy from the mandible mass showed diffuse amyloid materials denuded of cancerous cells. The differential diagnosis was a calcified odontogenic tumor, amyloidosis, amyloidoma, or plasmacytoma. The patient was referred to do a blood test of beta amyloid to ensure the amyloid circulating in the blood and related systemic diseases. Furthermore, a cone-beam computed tomography (CBCT) has been done, and it was shown later. The blood test was negative. No further urine test to detect the Bence Jones protein was done because the test was not available at the time of the diagnostic procedure, nor was serum protein electrophoreses performed, because there was no relevant clinical data for AL amyloidosis.

## 3. Radiology

### 3.1. Radiographic Examination

**Figure 2:**
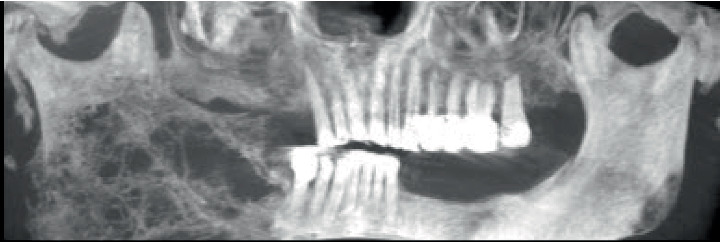
Reconstructed panoramic view generated from OnDemand3D App software (Cybermed Inc., Seoul, Korea) after reconstructing the computed tomography planes.

The patient complained of difficulty opening her mouth because of a multinodular mass in the right mandible extending above the sternocleidomastoid muscle, as presented in Figure 2. As indicated, a computed tomography (CT) scan was performed.

**Figure 3:**
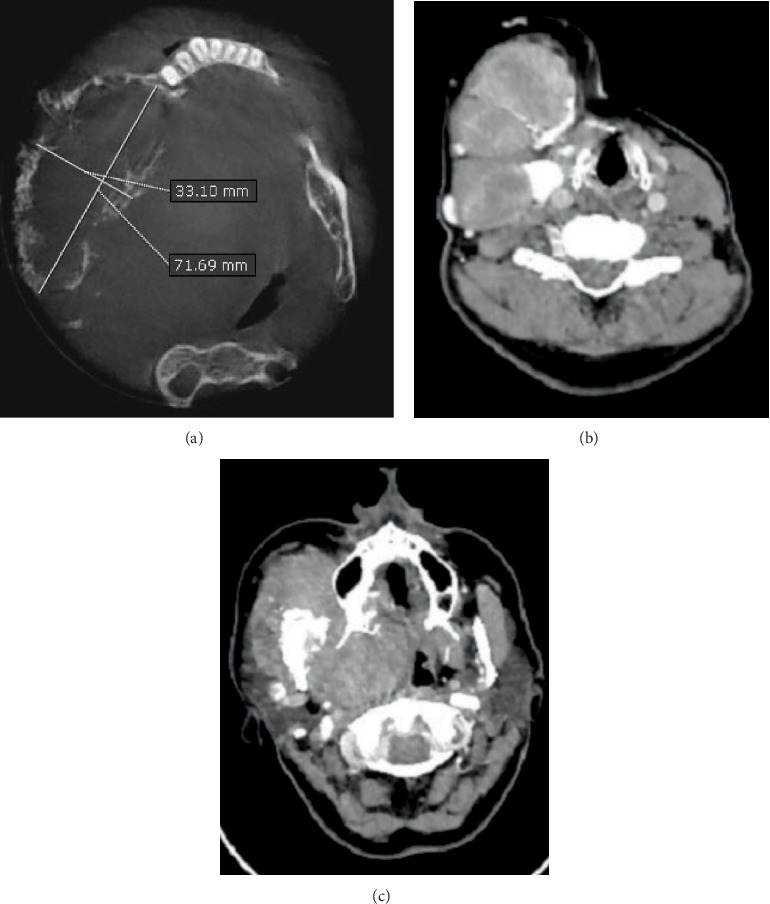
(a) Computed tomography axial plane generated by OnDemand3D App software (Cybermed Inc., Seoul, Korea) showing the anteroposterior dimension of the lesion and the buccolingual dimension. Raw computed tomography axial planes showing (b) three lobulated masses and (c) the invasion of the buccal space on the right side.

**Figure 4:**
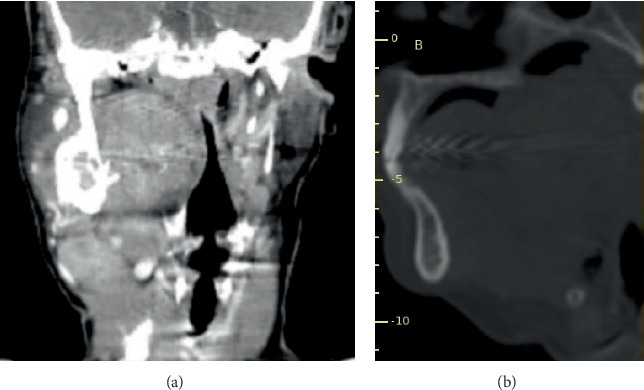
Severe airway obstruction shown on (a) computed tomography coronal plan and (b) sagittal cut.

**Figure 5:**
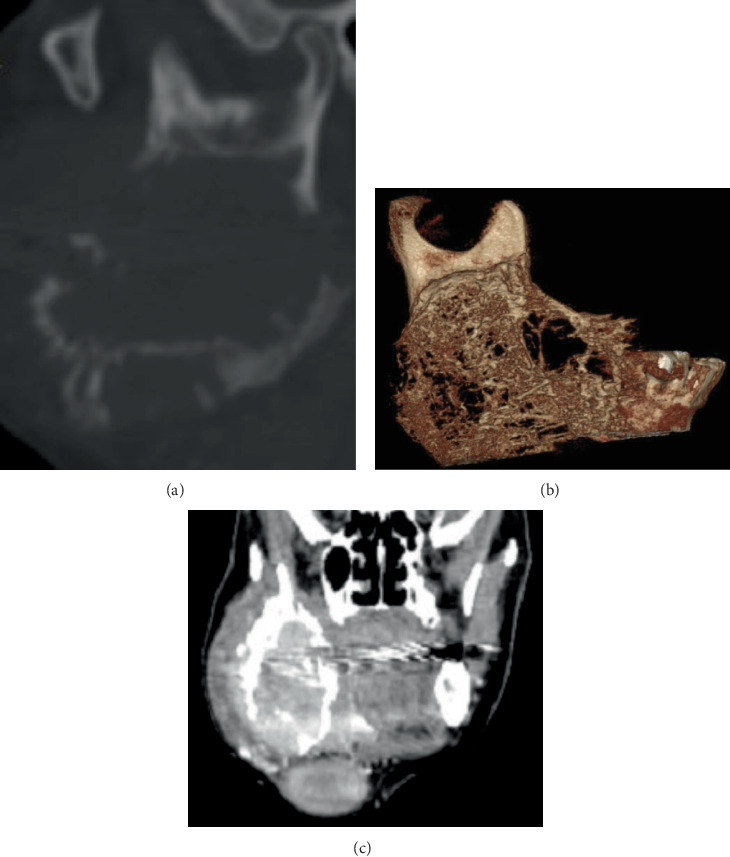
Superior extension of the lesion presented on (a) the sagittal computed tomography cut and (b) three-dimensional segmented right mandible view generated by OnDemand3D App software (Cybermed Inc., Seoul, Korea). (c) Inferior extension of the lesion presented on a raw coronal computed tomography plan.

After the raw axial cuts were reconstructed, the orthogonal planes were interpreted from head to toe twice, focusing on the area of interest as follows:

- Size: On the axial plane, the lesion measured 6.7 cm anteroposteriorly and 3.9 cm buccolingually, as illustrated in Figure 3.
- Internal structure: Extending inferiorly in the axial cuts, the lesion reveals three tiny, well-lobulated soft tissue masses, as illustrated in Figure 3.
- Margins: Its margins display a sunburst pattern indicative of malignancy, with numerous perforations in both the buccal and lingual bone plates.
- Effect on adjacent structures: This substantial mass induces significant buccolingual expansion into the buccal spaces, as illustrated in Figure 3. Furthermore, Figure 3 demonstrates a significant soft tissue invasion lingually, resulting in critical constriction of the airway space, which poses a life-threatening risk, as depicted also in the coronal and sagittal cuts in Figure 4.
- Location: The axial cuts revealed an ill-defined expansile multilocular osteolytic mass on the patient's right side. The lesion extends posteriorly from the condylar region to the distal aspect of the mandibular right second premolar region anteriorly. The coronal and sagittal planes indicate that the lesion grows superiorly from the subcondylar region, extending 1.5 cm beyond the sigmoid notch as presented in Figure 5, and descends inferiorly to encompass the entire mandibular ramus and body, reaching the inferior border of the mandible and further extending into the submandibular and carotid triangles, as highlighted in Figure 5.
- Bony changes: The mass is accompanied by erosive alveolar bone destruction with irregular borders.

A tentative diagnosis of intraosseous carcinoma in the right retromolar region was established based on clinical and radiological findings. However, the differential diagnosis list included osteosarcoma, lymphoma, and central giant cell lesion.

### 3.2. Surgical Procedure

Surgical technique: After radiological and clinical evaluation and due to the extensive nature of the tumor in the mandible, a decision was made to perform a total right hemimandibulectomy. General endotracheal anesthesia was administered via an orotracheal tube. Following skin preparation with iodine solution and marking of the incision line, lidocaine with 1:100,000 epinephrine was locally injected into the submental and submandibular areas. A Z-shaped lip-splitting incision was then made.

Further dissection was performed using cautery and forceps. During the procedure, hemostasis and vascular ligation were achieved using curved mosquito forceps and 3/0 absorbable Vicryl sutures. A full-thickness flap was elevated, exposing the right half of the mandible. A vertical bone cut was made at the right parasymphysis using a reciprocating saw with copious saline irrigation. The lower right first and second premolars, which were included in the tumor site, were removed.

The condyle was separated from the glenoid fossa, completing the total right hemimandibulectomy. The ablated portion of the mandible was found to be encapsulated. Additional dissection was performed to remove another encapsulated mass located in the right parapharyngeal space, which was excised. The maxillary artery was identified and preserved. Two masses in the submandibular triangle were also identified and removed. The facial artery and vein were carefully preserved.

The incision was extended into the neck region to allow exposure of the carotid triangle, where another mass was identified posterior to the internal jugular vein. The internal jugular vein was identified and preserved, and the mass was delicately removed.

Throughout the surgery, a drop in the hemoglobin level was noticed, and two units of blood were transfused to the patient.

Vascular ligation was continued after the total ablation to ensure hemostasis. A reconstruction plate was subsequently fixed using bicortical screws.

The muscular and subcutaneous layers were closed using simple interrupted sutures with 3/0 absorbable Vicryl. The skin was closed with simple interrupted 3/0 nylon sutures. Finally, a drainage tube was secured in place. All excised masses were submitted for comprehensive histopathological evaluation.

### 3.3. Histology

**Figure 6:**
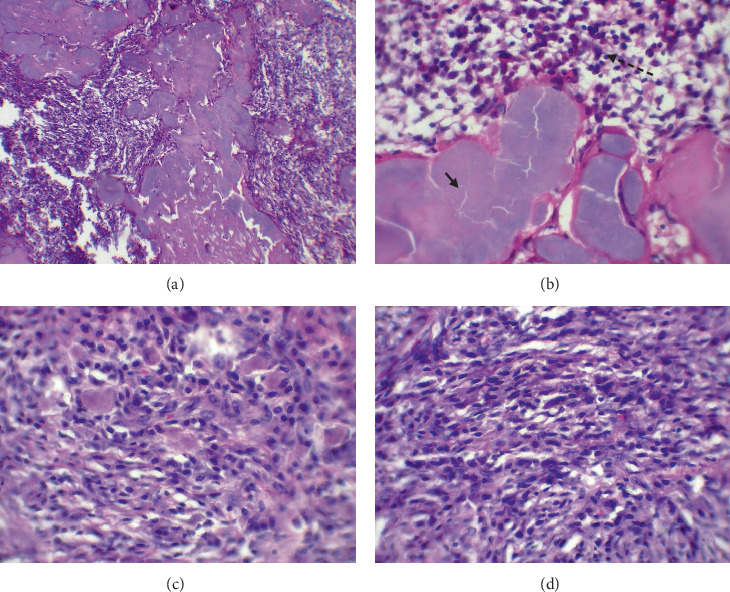
(a) A wide distribution of amyloid deposition within an infiltration of plasma cells (magnification ×100). (b) On higher magnification (×400), the amyloid (black arrow) and the cancerous plasma cell (dotted black arrow). (c, d) Foci of spindle cells revealed atypical changes among the plasma cells (magnification ×400).

**Figure 7:**
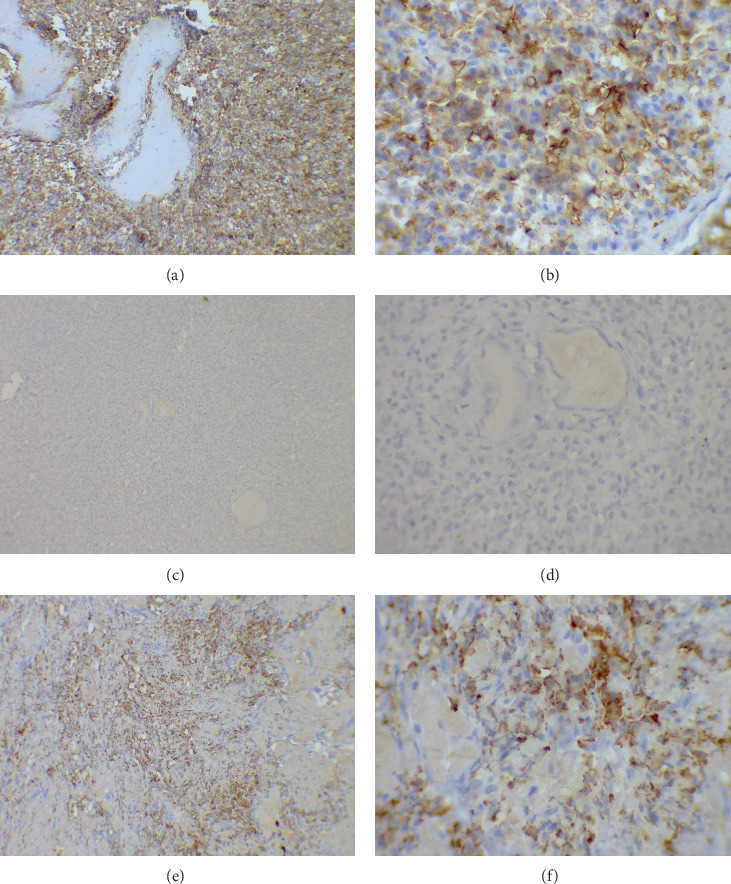
(a) Immunohistochemistry stains: CD138 expression in the tumors showing a diffuse strong positivity (magnification ×100). (b) Membranous strong positivity of CD138 in the tumor cells through the section (magnification ×400). Kappa and lambda light chain staining showed (c, d) the negativity in the kappa stain (magnification ×100; magnification ×400) and (e, f) the positivity restricted to lambda chain in the tumor cells (magnification ×100; magnification ×400).

The specimen received by the oral pathology lab in the faculty of dentistry was composed of a destructive right hemimandible and three mass and neck lymph nodes. All were immersed in formalin solution (diluted 10%). All masses were prepared and stained firstly in traditional stains (hematoxylin and eosin). The slides were studied under a light microscope. Examination of all slides revealed deposition of amorphous eosinophilic materials surrounded by clusters of atypical plasma cells. The mandible slides showed a wide deposition of amorphous eosinophilic materials with minimal infiltration of cancerous cells (Figure 6a,b). These materials were stained red by Congo red stain and appeared in apple-green birefringence by polarized light. The neck masses showed diffuse proliferation of plasmacytoid cells with occasional spindling and atypical nuclei (Figure 6c,d). The lymph nodes were well delineated and involved by the mentioned proliferation and deposition. Immunohistochemical staining revealed positivity for CD138 (Figure 7a,b), lambda light chain restriction (Figure 7c,d), and diffuse positivity for lambda (Figure 7e,f). The final diagnosis was plasmacytoma with amyloid deposition.

### 3.4. Follow-Up

The patient is now under periodic follow-up for 1 year. There are no clinical symptoms or signs of recurrence. The patient is in good health functionally.

## 4. Discussion

Plasmacytoma is a malignant tumor of white blood cells. It is divided into solitary and extramedullary types. The solitary type is seen mainly in the bone marrow of vertebrae and long bones. While the extramedullary occurs usually in the head and neck region and rarely involves the bone marrow [12, 13]. Still, the plasmacytoma is an extremely rare entity in the gnathic bones and represents nearly 4.4% of all solitary plasmacytomas, almost always in the mandible rather than the maxilla. The diagnosis is made by confirming the infiltration of plasmacytoid cells only on the mandible and no other bone. It is most commonly seen in middle-aged patients around 6 decades old and more relevant in men than in women. The clinical presentations manifest in face swelling because of expansion of the cortical bones. Tooth dislocation or, rarely, root resorption may be seen. In addition to tooth mobility, taste and sensory disturbances are reported in some cases [14, 15].

The radiographic findings of the present plasmacytoma case correspond with the existing literature. Malignant plasma cells release osteoclast-activating substances that induce osteoclasts to resorb bone; thus, radiographic examinations typically show osteolytic lesions marked by unilocular or multilocular radiolucency. Lae et al. performed a clinicopathologic study of 33 instances of myeloma involving the jawbones. Three radiographic manifestations of plasmacytoma were identified: multilocular soap bubble lesions, unilocular radiolucency mimicking a cyst, and poorly defined destructive bone resorption. A poorly delineated radiolucency with considerable cortical destruction of the whole posterior jaw was noted in the present case [16].

The immunohistochemistry (IHC) stain is essential in making a final diagnosis of plasmacytoma. CD138 is a role stain in the process of diagnosis as it shows the abnormal plasmacytoid proliferation. In addition, LCA and EMA could be supportive stains to be used in the IHC panel. Kappa and lambda light chains also play an essential role in diagnosis; in nonneoplastic proliferation, neither kappa nor lambda is restricted. While in neoplastic proliferation, either kappa or lambda is restricted [17].

Amyloid deposition in plasmacytoma is a rare occurrence, occurring in approximately 15% of EMPs, suggesting a potential relationship that warrants further investigation. Amyloid fibrils can be found alongside plasmacytoid cell proliferation. In MM, amyloid deposits may manifest as localized amyloidoma or be accompanied by EMP. Diagnosis typically involves histological examination; Congo red staining is used to identify amyloid deposits. In plasmacytoma, the amyloid deposition can complicate the clinical features, often indicating a poor prognosis, especially in cases of MM [18]. In literature, focus is directed toward the link between amyloidosis deposition and EMP in order to help in developing targeted therapies and improving patient outcomes [19].

Local excision of the tumor may be recommended as the first choice of treatment. This approach is particularly relevant for patients with localized amyloidosis associated with plasmacytoma, as the complete removal of the tumor can help manage both conditions. Although in EMPs, the radiotherapy approach may be the excellent choice, as the spreading of the tumor may prevent the possibility of the surgery. While not the primary treatment for localized plasmacytoma, chemotherapy may be considered in cases where there is a risk of progression to MM or if the disease is more aggressive. This is more common in patients with systemic involvement or those who do not respond to radiation or surgery. If localized amyloidosis is present, treatment may also focus on managing the amyloid deposits. This can include monitoring for systemic involvement and addressing any symptoms related to amyloid deposition in affected organs. Regular follow-up is crucial for patients treated for EMPC, especially to monitor for recurrence or progression to MM. The prognosis for localized plasmacytoma is generally better than for MM, with a 10-year survival rate of approximately 55% [20, 21].

## 5. Conclusion and Future Direction

Plasmacytoma with potential amyloid deposition could influence clinical practices, encouraging more comprehensive diagnostic approaches and tailored treatment strategies, especially in cases where amyloid is suspected. Localized plasmacytoma with amyloid deposition may have a different prognosis compared to systemic amyloidosis or MM. Future research could focus on long-term outcomes and survival rates in similar cases, contributing to the understanding of disease progression and management. The characterization of the amyloid protein may open avenues for developing new therapeutic strategies aimed at targeting the specific light chains involved in amyloid formation, potentially leading to more effective treatments for patients with localized amyloidosis and plasmacytoma.

## Data Availability

The data that support the findings of this study are available on request from the corresponding author. The data are not publicly available due to privacy or ethical restrictions.
